# Effect of implant‒abutment connections on abutment screw loosening: An in vitro study

**DOI:** 10.34172/joddd.29883

**Published:** 2024-03-29

**Authors:** Feridoun Parnia, Amin Nourizadeh, Elnaz Shafiee

**Affiliations:** ^1^Department of Prosthodontics, Faculty of Dentistry, Tabriz University of Medical Sciences, Tabriz, Iran; ^2^Department of Prosthodontics, Faculty of Dentistry, Islamic Azad University of Tabriz, Iran

**Keywords:** Dental implants, Dental implant‒abutment connection

## Abstract

**Background.:**

The widespread use of dental implants as a predictable treatment choice has drawn attention to their complications as a major challenge despite their high clinical success rates. In this context, loosening of the abutment screw in posterior single crowns is the most common problem; the use of adequate preload and proper anti-rotational features at implant‒abutment interface appear to be two main solutions to such a problem. The present study evaluated the effect of implant‒abutment connections in four different implant systems before and after cyclic loading.

**Methods.:**

Intra-Lock, Dentis, Xive, and Dio implant systems were used in this study. Each system underwent one million cycles of dynamic forces eight times with a magnitude of 110 N. For each specimen after tightening the screw with a torque of 32 Ncm, the detorque values were measured and recorded by a digital torquemeter after and before cyclic loading. Data were analyzed by Kolmogorov-Smirnov, Levene’s, one-way ANOVA, and post hoc Tukey tests.

**Results.:**

Initial detorque values between the study groups showed significant differences (*P*<0.0001). Pairwise comparisons showed significantly lower primary detorque values in the Dentis system compared to the three other systems (*P*<0.0001). After cyclic loading, significant differences were observed between the study groups (*P*<0.0001). Pairwise comparisons of the groups showed significant differences between all the systems after loading.

**Conclusion.:**

The type of implant‒abutment connection is an essential factor influencing the amount of abutment screw loosening.

## Introduction

 Although implant treatments have a generally high success rate, the prosthetic and surgical complications of implant-supported prosthetic appliances are not uncommon.^1^ Prosthetic complications can be classified as fracture of the veneer, loosening of the abutment screw, fracture of the prosthetic screw, and fracture of the metallic framework and implant, of which loosening of the abutment screw in posterior single crowns is the most common complication with an incidence rate of 12.7%.^2,3^ Reasons for loosening of the abutment screw include inadequate preload, inappropriate implant position, inappropriate occlusal or anatomic design of the crown, variations in hex dimensions, poor adaptation of implant components, poor design of the screw, excessive occlusal load, and poor anti-rotational features,^4-6^ of which inadequate preload and poor anti-rotational features have the most significant effects on prevention of such a problem.^7-15^

 Preload, as an important property involved in the prevention of loosening of the abutment screw, is defined as a tensile force produced in the abutment screw as a result of its tightening.^7,16-18^ This screw tightening results in an elongation in the structure of the screw, the elastic recovery of which condenses the different components of the implant and abutment together, keeping them next to each other, referred to as the clamping force.^9^ The amount of this force is primarily dependent on the amount of torque applied, and some other factors, including the material type, design, and form of the screw head and its threads and surface roughness, affect it, too.^7,8^ If the amount of separating force exerted on the system does not exceed the amount of the clamping force, the implant‒abutment connection will remain stable.^8^

 Another critical factor in preventing abutment screw loosening is its proper anti-rotational features. Since Branemark introduced the external hex as an anti-rotational feature and with an increase in the use of implant-supported prosthetic appliances to replace single teeth, this feature has come to attention more than ever.^19,20^ Considering the high incidence rate of screw loosening in implant systems with external hex and the inefficacy of this type of connection in preventing abutment screw loosening,^21^ different types of anti-rotational features have been introduced,^22^ which are classified into butt-joint and conical connection groups.^23^ Both these groups are further divided into subclasses based on the presence or absence of locking against rotation or detachment of the abutment.^23^

 Butt-joint connections are composed of two surfaces located perpendicular to the implant axis and joined together by the screw. In such connections, there is a small space between the components; therefore, they are called slip-fit connections.^24^ In contrast, conical connections have a frictional fit with a tight connection between the implant and abutment.^25^

 Emphasis on anti-rotational features in implant‒abutment connections in the prevention of abutment screw lessening is so strong that, at present, more than 20 different types of these connections are used in various implant systems.^26^ Considering the significant number of studies indicating the lower effect of external connections compared to internal ones, there is currently more focus on internal connections. Binon reported a direct relationship between the rotational misfit and abutment screw loosening in implants with external hex.^14^ Mollersten clearly showed that internal connections have better efficacy in maintaining connection stability without abutment screw loosening compared to external ones.^27^ Only a limited number of studies have evaluated the efficacy of different internal connections; for example, a study by Norton showed a higher efficacy for conical connections compared to hexagonal connections.^28^ Since the anti-rotational feature has a vital role in preventing abutment screw loosening, especially in replacing single teeth, and also given that there is a very wide range of designs for implant‒abutment connections, it is necessary to compare the efficacy of these designs.

 The present study evaluated the effect of implant‒abutment connections in four different implant systems before and after cyclic loading.

## Methods

 In the present study, abutments, fixtures, and abutment screws of four implant systems, including Intra-Lock (Intra-Lock International, Florida, USA) with notch-shaped internal connection, Dentis (Cleanlant Implants, Seoul, Korea) with the morse taper plus hexagonal internal connection, Xive (Friadent, Dentsply Co., Mannheim, Germany) hexagonal internal connection, and Dio (Dio Implants, SMTorx/Extrawide, Busan, Korea) with torx (star-shaped) internal connection, were used to evaluate the effect of the type of implant‒abutment connection on abutment screw loosening. Table 1 presents the lengths and diameters of the implants and the abutments selected.

**Table 1:** The list of the components used in the present study

**Implant type**	**Connection type**	**Fixture diameter**	**Fixture length**	**Abutment diameter**	**Abutment length**	**Gingival** **height**
Xive	Internal Hex	4.5	11	4.5	5	1
Intra-Lock	In-Dex	4.3	11.5	5	5	1
Dentis	Internal Hex	4.3	12	4.5	5	1
Dio	Totx	4.5	12	4.8	5	1

 Under the existing limitations, an attempt was made to use fixtures with almost similar lengths and diameters and abutments with almost similar heights, diameters, and gingival heights to eliminate confounding factors. Eight fixtures from each implant system were mounted in polypropylene cylinders measuring 30 mm in length and 10 mm in diameter using self-cured acrylic resin (SR Triplex, Cold, Ivoclar Vivadent Schaan, Liechtenstein). The internal surfaces of the cylinders had been roughened to provide more retention and better connection of the acrylic resin. In addition, the cylinders had a transverse hole for placing a horizontal pin to prevent rotation during the application of tightening and loosening torques. Then the abutments selected for each system were screwed on the fixtures using a TQ 800 digital torquemeter (Lutron Digital Instruments, Taipei, Taiwan) at 32-Ncm torque. In the next stage, metallic crowns resembling mandibular first molars, with external anatomic dimensions, which had been manufactured using a single template, were cemented using Temp Bond NE (Kerr Corporation, California, USA) temporary cement by one operator following manufacturer’s instructions; the crowns had an access hole on the occlusal surface for easy access of torquemeter and measurement of detorque values.

 After the samples were prepared and before cyclic loading, the samples were fastened on a table vice to measure initial detorque values, which were recorded for each system after tightening with a torque of 32 Ncm. The procedure was repeated eight times for each system under study. In the next stage, the samples were placed in a piece of cyclic loading equipment (SD Mechatronik Chewing Simulator CS4, Germany) after the screws were tightened again using 32-Ncm torque with a digital torquemeter and cementation of the fabricated crowns; the samples were fixed in the chamber of the equipment with self-cured acrylic resin. It should be pointed out that the equipment can accommodate two samples at the same time. In the next stage, each sample underwent 1×10^6^ cycles of load, with the magnitude of 110 N^29^ perpendicular to the occlusal surface of the crown at a frequency of three cycles/second, corresponding to 40 months of clinical use.^26^ During the loading steps and after every 100 000 cycles, the samples were evaluated concerning screw loosening, cement failure, acrylic resin fracture, and other failures.

**Figure 1:**
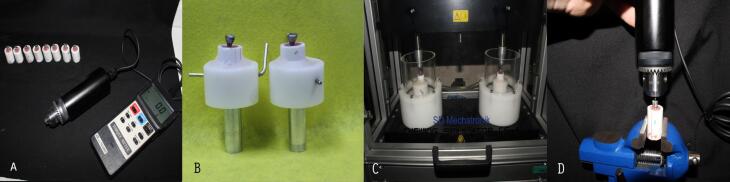
A: Prepared samples and digital torquemeter. B: Samples mounted to be placed in the cyclic loading machine. C: Samples in the cyclic loading machine. D: Measurement of detorque value.

 After completing load cycling procedures, the detorque values were measured and recorded by a digital torquemeter. After measurement procedures ended for the samples loaded, cyclic loading procedures were repeated for each sample. Therefore, each system underwent the test procedure eight times (Figure 1).

 After measuring and registration of detorque values before and after cyclic loading of each sample, descriptive data (means and standard deviations) were calculated. Kolmogorov-Smirnov test was used to evaluate the normality of data. Levene’s test was used to evaluate the homogeneity of variances between the groups. One-way ANOVA was used to evaluate and compare torque values between the groups. Tukey post hoc tests were used for pairwise comparisons of the groups. SPSS 17 was used for statistical analysis. Statistical significance was defined at *P* < 0.05.

## Results

 After measuring initial torque values and completing cyclic loading procedures, detorque values were measured and recorded for each specimen. No screw loosening, cement failure of crowns, fracture of acrylic retainers of fixtures, detachment of acrylic retainer of polypropylene cylinders, etc. were observed. Table 2 presents descriptive data of initial detorque values measured in the study groups.

**Table 2:** Descriptive data of initial detorque values in Ncm

**Implant system**	**Mean (SD)**	**95% confidence interval for mean**		**Pairwise comparisons** **(*P* value)**
		**Lower**	**Upper**	
Xive	29.8250 (1.40687)	28.6488	31.0012	Dio (0.811) Dentis (0.000) Intra Lock (0.580)
Intra Lock	30.6750 (1.15974)	29.7054	31.6446	Dio (0.156) Dentis (0.000) Xive (0.580)
Dentis	25,2375(1.40605)	24.0620	26,4130	Dio (0.000) Xive (0.000) Intra Lock (0.000)
Dio	29.2375 (1.30815)	28.1439	30.3311	Dentis (0.000) Xive (0.811) Intra Lock (0.156)

**Figure 2:**
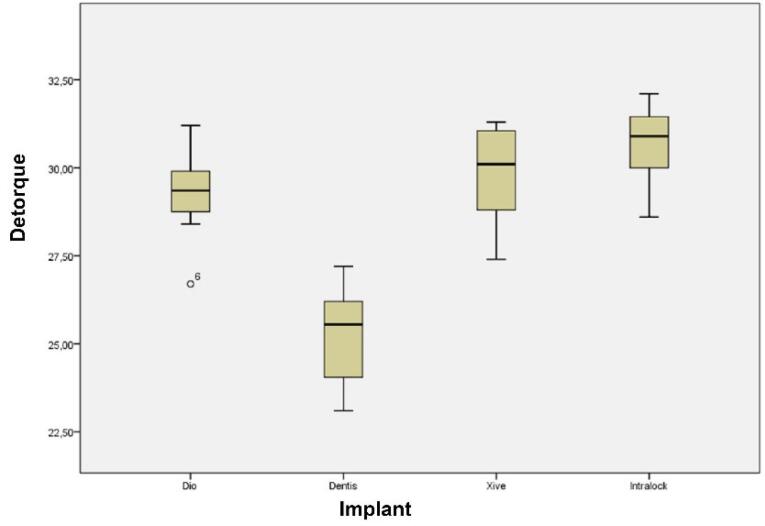
Box-plot of primary detorque values of different implant systems.

 The maximum and minimum initial detorque values were recorded in the Intra-Lock and Dentis systems, respectively. Kolmogorov-Smirnov test showed a normal distribution of initial detorque data between all the groups (*P* = 0.200). Levene’s test showed homogeneity of variances in all the groups (*P* = 0.830). Therefore, all the prerequisites for one-way ANOVA were available to compare initial detorque value means. ANOVA showed significant differences between the study groups (*P* = 0.000). Pairwise comparisons of initial detorque values between the groups with post hoc Tukey tests showed significantly lower primary detorque values in the Dentis system compared to the three other systems (*P* = 0.000). There were no statistically significant differences between the Xive, Intra-Lock, and Dio systems (Figure 2). Table 3 presents the descriptive data of detorque values measured after cyclic loading. The maximum and minimum primary detorque values after cyclic loading were recorded in the Intra-Lock and Dentis systems, respectively.

**Table 3:** Descriptive data of detorque values in Ncm after cyclic loading

**Implant system**	**Mean (SD)**	**95% Confidence interval for mean**		**Pairwise comparisons (*P* value)**
		**lower**	**upper**	
Dentis	20.8250 (0.9808)	20.0050	21.6450	Dio (*P* = 0.000)
				Xive (*P* = 0.000)
				Intralock (*P* = 0.000)
Dio	24.4250 (1.3853)	23.2668	25.5832	Dentis (*P* = 0.000)
				Xive (*P* = 0.023)
				Intralock (*P* = 0.000)
Xive	26.6000 (1.8792)	25.0289	28.1711	Dentis (*P* = 0.000)
				Dio (*P* = 0.023)
				Intralock (*P* = 0.007)
Intralock	29.1125 (1.2822)	28.0405	30.1845	Dentis (P = 0.000)
				Dio (*P* = 0.000)
				Xive (*P* = 0.007)

**Figure 3:**
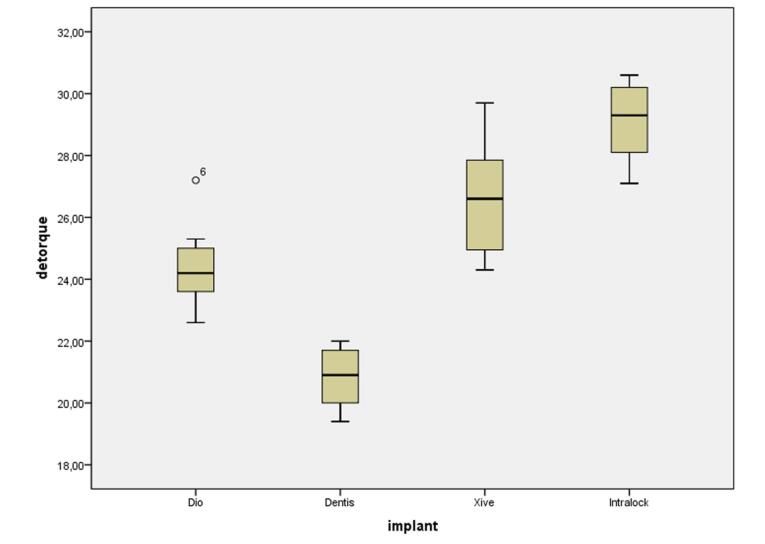
Box-plot of detorque values of different implant systems after cyclic loading.

 One-way ANOVA was used to compare the mean detorque values after cyclic loading due to the normal distribution of data as shown by the Kolmogrov-Smirnov test (*P* = 0.200) and homogeneity of variances as shown by Levene’s test (*P* = 0.269); one-way ANOVA showed significant differences between the study groups (*P* = 0.000). Pairwise comparisons of the groups showed significant differences in the mean detorque values after cyclic loading between all the systems under study, with the highest detorque values in the Intra-Lock system after cyclic loading, followed by the Xive and Dio systems; the Dentis system exhibited the least detorque values after cyclic loading (Figure 3).

## Discussion

 The widespread use of dental implants as a predictable treatment option has drawn attention to its complications as a significant challenge despite its high clinical success rates. In this context, loosening of the abutment screw in posterior single crowns is the most common problem; the use of adequate preload and proper anti-rotational features at the implant‒abutment interface appear to be two main solutions to such a problem.^1-3,5,6,30^

 Only a limited number of studies have evaluated the frequency and amount of abutment screw loosening in different implant‒abutment connection types, and different ranges have been reported. A common consideration in all these studies is the higher incidence of screw loosening in external hex connection types,^26^ which might be attributed to the limited engagement between the anti-rotational components in such connections.^31,32^ Different internal connections between implants and abutments have been introduced to overcome this problem.^33^ Such connections result in better distribution of lateral forces within the implant body, preventing transmission of excessive forces to the abutment screw. In addition, a proper internal connection leads to integration between the implant and abutment, preventing the loosening of the connection.^33^ In the present study, the amount of abutment screw loosening was compared between four types of internal connection.

 All the systems under study exhibited a decrease in initial detorque values even before loading. In this context, after tightening the screws with 32-Ncm torque, the detorque values with which all the systems were immediately loosened were less than the initial amount.

 It might be possible to explain such a decrease in the amount of initial torque by Bickford’s theory,^17^ which explains the mechanisms involved in fractures at connection sites. Based on this theory, the detorque value is a proportion of the initial torque (preload) which has been preserved in the abutment screw. In cases in which there is some misfit at contact surfaces a proportion of the torque preserved in the screw is lost. The decrease in the initial torque preserved in the screw can be influenced by the abutment screw material and the precision of fabrication of components that come into contact with each other. Therefore, the screw connection will be more stable with an increase in the precision of connection between the implant components.^14^ In the present study, the differences in initial torque values between the systems evaluated were statistically significant; in this context, the Intra-Lock system exhibited the least decrease in the initial torque with 4.4%, followed by the Xive system with 6.9%, the Dio system with 8.8% and the Dentis system with 21.2%.

 The high initial detorque values in the Intra-Lock, Xive, and Dio systems might be attributed to the precise connection between different components at the connection site, preventing the loss of the torque produced in the screw. On the other hand, the cold welding phenomenon at the connection site might be a potential factor in preventing the decrease in initial detorque.^26^ However, Norton^34^ studied initial detorque values and concluded that cold welding occurs only in high torques (higher than 100 Ncm). The incidence of this phenomenon appears to be less effective in preventing the decrease in initial detorque values. After applying 1 million dynamic cyclic loads on the specimens, the specimens’ detorque values were significantly different. The highest detorque value after cyclic loading was recorded in the Intra-Lock system with 90.9% of the initial torque. After exerting dynamic forces, the Xive, Dio, and Dentis systems preserved 83.1%, 76.2%, and 65% of their initial torque values, respectively. Only a limited number of studies have evaluated the effect of the type of implant‒abutment connection on abutment screw loosening. Ha et al^26^ studied the impact of internal connection and abutment diameter on the abutment screw loosening. Six implant systems with different connections and small and large diameters were used. The results did not reveal significant differences in the initial detorque values between various implant systems. However, after 1 million cycles of dynamic loading, the detorque values were significantly different between the different systems, with the highest detorque values before and after cyclic loading in the SS II (Osstem) system, which has Morse Taper Octa connection type with an angle of 8°. According to Ha et al,^26^ all the implant systems exhibited a decrease in detorque values before and after cyclic loading except for the SS II system, which demonstrated an increase in detorque values after cyclic loading. The authors attributed this increase in detorque values to the properties of the connection, which was tapered. In this type of connection, the forces applied encounter resistance at the tapered interface, and a lock form and frictional resistance are formed at the connection site so that the abutment screw is protected against these forces to a great degree. Another justification for an increase in detorque values after cyclic loading is cold welding at 8° Morse taper connection type, contrary to Norton’s claim,^34^ attributing the phenomenon to high-torque situations.

 An important consideration in Ha and colleagues’^26^ study was that in the SS II system, a solid abutment was used, but in other systems evaluated, two-piece abutments were used. It appears the mechanism and the amount of force applied to the abutment screw are different in the two-piece versions compared to the solid ones, possibly explaining differences in the results of that study. The connections used in that study were of hex, Morse taper, and Trican types; however, in the present study, the connections were of Torx, In-Dex, and hex types. Despite differences in the designs of the two studies, the amount of decrease in initial torques is almost similar. After applying dynamic forces, except for the SS II system, which exhibited an increase in detorque values, the detorque values between the two studies were almost similar. In a study by Khraisat et al.^35^ on the effect of cyclic loading on abutment screw loosening, five Branemark Mark IV implants (Nobel Biocare) were used, which measured 4× 10 mm in dimensions with external hex connection. The results showed a mean initial detorque value of 24.5 Ncm after tightening the screws with a torque of 32 Ncm, which is less than the initial detorque values measured in the present study. In that study, the detorque value was 20.2 Ncm after applying 1 million dynamic load cycles, which is again less than that in the present study. The differences might be attributed to the type of connections used in the two studies. In the external connection, no lock system is created against the forces applied, and all the forces applied are transferred to the abutment crew; however, in internal connections, a fraction of the force applied is neutralized at the connection interface by some mechanisms, such as positive lock form and frictional retention.^36^

 Based on the results of the present study, it appears the type of implant‒abutment connection is a factor affecting the amount of abutment screw loosening. In addition, precise and proper fitting of components coming into contact at the connection affects the amount of abutment screw loosening before and after force application; the fit is in itself under the influence of the quality of fabrication and the materials used. However, it should be remembered that despite a decrease in detorque values after applying dynamic forces, all the systems under study had some torque sufficient for proper clinical efficacy.

## Conclusion

 The results of the present study showed that the type of implant‒abutment connection is an important factor influencing the amount of abutment screw loosening. Due to precise manufacturing and the shape of the connection, the Intra-lock system had the highest detorque values, and the Dentis system had the lowest. All the systems showed clinically acceptable results after one million dynamic cycles.

## Competing Interests

 The authors declare no conflict(s) of interest related to the publication of this work.

## Ethical Approval

 Not applicable.

## Funding

 None.
